# SIRT-3 Modulation by Resveratrol Improves Mitochondrial Oxidative Phosphorylation in Diabetic Heart through Deacetylation of TFAM

**DOI:** 10.3390/cells7120235

**Published:** 2018-11-28

**Authors:** Pankaj K. Bagul, Parmeshwar B. Katare, Paramesha Bugga, Amit K. Dinda, Sanjay K. Banerjee

**Affiliations:** 1 Drug Discovery Research Center (DDRC), Translational Health Science and Technology Institute (THSTI), Faridabad 121001, India; pankajbagul2787@gmail.com (P.K.B.); Parmeshwar@thsti.res.in (P.B.K.); bparamesha@thsti.res.in (P.B.); 2 Department of Pathology, All India Institute of Medical Science (AIIMS), New Delhi 110029, India; akdinda@hotmail.com

**Keywords:** diabetic cardiomyopathy, transcription factor A, mitochondria, acetylation

## Abstract

Background and Purpose: Mitochondrial dysfunction remains the crucial cause for many heart diseases including diabetic cardiomyopathy (DCM). Sirtuin-3 (SIRT-3) is a protein deacetylase localized in the mitochondria and regulates mitochondrial function. Being a noteworthy mitochondrial protein deacetylase enzyme, the role of SIRT-3 in DCM is yet to be explored. Experimental Approach: Diabetes mellitus (Type-I, T1DM) was induced using streptozotocin (STZ, 50 mg/kg) in male Sprague Dawley (SD) rats. Rats with >200 mg/dL blood glucose levels were then divided randomly into two groups, DIA and DIA + RESV, where vehicle and resveratrol (25 mg/kg/day) were administered orally in both groups, respectively. Cardiac oxidative stress, fibrosis, and mitochondrial parameters were evaluated. H9c2 cells were transfected with SIRT-3 siRNA and shRNA, and ORF plasmid for silencing and overexpression, respectively. Key Results: After eight weeks, diabetic rat heart showed reduced cardiac cell size, increased oxidative stress and reduction of the activities of enzymes involved in mitochondrial oxidative phosphorylation (OXPHOS). There was reduced expression and activity of SIRT-3 and mitochondrial transcription factor (TFAM) in diabetic heart. Reduced SIRT-3 expression is also correlated with increased acetylation, decreased mitochondrial DNA (mtDNA) binding activity of TFAM, and reduced transcription of mitochondrial DNA encoded genes. Administration of resveratrol prevented the decrease in SIRT-3 and TFAM activity, which was corresponding to the reduced acetylation status of TFAM. Silencing SIRT-3 using siRNA in H9C2 cells showed increased acetylation of TFAM. Conclusion and Implications: Together our data shows that resveratrol activates SIRT-3, regulates the acetylation status of TFAM and preserves the mitochondrial function along with cellular size in diabetic rat heart.

## 1. Introduction

Sirtuins are the class III histone deacetylase enzymes (HDAC) which are evolutionary conserved and possess NAD^+^ dependent deacetylase activity. Among the seven different sirtuins, SIRT-3, SIRT-4, and SIRT-5 are mostly localized in the mitochondria. Growing evidence suggest that SIRT-3 has a crucial role in regulating metabolic homeostasis through multi-tissue coupling [1]. Reports confirmed SIRT-3 as a major protein deacetylase in mitochondria and thus explain its importance in energy metabolism. SIRT-3 is involved in regulating cellular redox status, mitochondrial energetics, biogenesis, dynamics, and apoptosis [2,3,4,5]. Cardiac mitochondrial proteins are three times more acetylated than other tissue proteins [6]. Cardiac complications remain the major cause of mortality in diabetes and explain the urgent need for mitigation. Looking at the basics of cardiac metabolic remodeling, the robust nature of cardiac cells to shift the substrate utilization based on demand is regulated by protein acetylation or deacetylation [7]. Cardiac complications in diabetes showed altered mitochondrial energetics, dysregulation of electron transport chain (ETC) complex, electron leak, and superoxide generation [8].

Mitochondrial DNA transcription encodes thirteen proteins for ETC complex assembly is regulated by TFAM, a mitochondrial transcription factor. All these thirteen proteins are essential to form a functional assembly with the nuclear encoded proteins of ETC complex. Thus, it is not surprising that altered transcriptional activity of mtDNA affects the capacity of OXPHOS. Most of the proteins/enzymes enrolled in the metabolic pathways are regulated by acetylation. Therefore, we hypothesized that altered acetylation balance in the diabetic heart may also affect mtDNA transcription.

Targeting mitochondrial energy metabolism pathways for the mitigation of disease pathology is the focus of today’s drug discovery. Nowadays, sirtuins are considered as emerging druggable targets for multiple diseases including diabetes [9]. Hebert and colleagues reported that the absence of SIRT-3, a mitochondrial sirtuin, increased the acetylation modification of mitochondrial protein almost two-fold [10]. In addition, it has been shown that SIRT3 enhances mitochondrial respiration rate and ATP production by coordinated activation of mitochondrial metabolic pathways and ROS detoxification [11,12]. Recent study by Zhang and colleagues showed that resveratrol glucoside activates SIRT-3 and attenuates DCM by upregulating autophagy and improving mitochondrial function [13].

Deliberating the role of SIRT-3 in mitochondrial energetics and mitochondrial dysfunction in diabetes, we thought to find out the missing link between SITR3 and mitochondrial function in the diabetic heart. We hypothesized that SIRT-3 has a crucial role in regulating the mitochondrial function in diabetic heart through regulating TFAM and thus targeting SIRT3 could be a useful approach to overcome cardiac complications in diabetes. In the present study, we have shown that resveratrol, which directly or indirectly can activate SIRT-3 [3,14,15] reduces acetylation of TFAM and rescued the mitochondrial dysfunction in the diabetic heart.

## 2. Materials and Methods

### 2.1. Animals

All the experiments were performed in accordance with relevant guidelines and regulations of the Institutional Animal Ethical Committee (IAEC) and Indian Institute of Chemical Technology (IICT), Hyderabad which is consistent with the Committee for the Purpose of Control and Supervision of Experiments on Animals (CPCSEA), New Delhi, India, guidelines. Male SD rats weighing 200 to 220 g and 8–10 weeks old were purchased from the National Institute of Nutrition (NIN), Hyderabad, India. Diabetes was induced by single intraperitoneal (i.p.) injection of 50 mg/kg STZ prepared in citrate buffer, pH 4.5; control rats received a similar volume of citrate buffer, pH 4.5 (i.p.). Rats were monitored for the development of hyperglycemia for the next seven days. Rats with stable increased blood glucose levels of more than 200 mg/dL were divided randomly into two groups: DIA group and DIA + RESV group (*n* = 12/group). Resveratrol was orally administered at a dose of 25 mg/kg/day, as per the previous study [16]. After eight weeks, rats were sacrificed and heart tissue was collected and stored in appropriate condition for downstream analysis.

### 2.2. Cell Culture

Rat cardiomyoblast cells (H9C2) were purchased from ATCC (Manassas, VA, USA) and cultured in Dulbecco’s modified Eagle’s medium (DMEM). The culture was supplemented with 10% fetal bovine serum and 100 μg/mL penicillin/streptomycin. After reaching 50 to 60% confluence, cells were treated with the respective siRNA for SIRT-1 and SIRT-3 knockdown, and ORF plasmid for SIRT3 overexpression (Dharmacon, USA) using dharmafect transfection reagent (Dharmacon, Lafayette, CO, USA) as per the manufacturer’s protocol. After 48 h of transfection, cells were washed and lysed for either protein or mRNA analysis. Isolated protein or mRNA was used for downstream analysis. Similarly, the stable SIRT-3 knockdown H9C2 cell line was developed with lentiviral vector shRNA (Dharmacon, USA) and used for gene expression analysis.

### 2.3. Serum Biochemical Analysis

Serum samples collected from rats were analyzed with auto blood analyzer (Siemens, Qulin, MO, USA) for the measurement of triglyceride, uric acid, HDL, creatinine, cholesterol, and SGOT. Measurement of serum insulin and glycated hemoglobin (HbA1c) was carried out using Mercodias’ rat insulin ELISA kit and Biosystems’ glycated hemoglobin detection kit, respectively.

### 2.4. Measurement of Cardiac Cell Size and Histopathological Examination

Heart weight to tail length ratio was evaluated at the end of the study to demonstrate cardiac phenotypic changes. Heart tissue samples fixed in neutral buffer formalin (10%) were paraffin-embedded for histopathological analysis. Sections (5-µm) were stained with hematoxylin and eosin and masons trichrome for further analysis. Cardiac cell size was measured using Image J as described previously [17,18].

### 2.5. Isolation of Mitochondria 

Mitochondria were isolated from equal weight of heart tissues from all groups using mitochondria isolation kit (Pierce, Thermo scientific, cat No: 89801). Isolated mitochondria were lysed and stored for downstream application.

### 2.6. Preparation of Heart Tissue Homogenate

Heart tissues were homogenized with ten times volume of 0.05 M phosphate buffer (pH 7.4) and centrifuged at 15,000× *g* for 30 min at 4 °C. Supernatant was stored at −80 °C for downstream analysis.

### 2.7. Measurement of Cardiac ROS, Antioxidant Levels and Oxidative Stress Parameters

The cardiac thiobarbituric acid assay (TBARS), reactive oxygen species (ROS), 2,2-diphenyl-1-picrylhydrazyl (DPPH), catalase, and superoxide dismutase (SOD) activity assays were conducted using protocol described by us previously [19,20,21,22].

### 2.8. Electron Transport Chain Complex Assembly Activity

Enzyme activity of citrate synthase and β-hydroxyl acyl-CoA dehydrogenase was measured according to the protocol described before [23]. The specific enzymatic activity of mitochondrial ETC complex I (NADH-ubiquinone oxidoreductase), complex II (succinate-ubiquinone oxidoreductase), and complex IV (cytochrome c oxidase) was measured as described previously [24]. ATP levels were measured using luminescence based assay (Life Technology Ltd. Waltham, MA, USA).

### 2.9. Gene Expression Analysis

Isolation of RNA from the heart tissue (*n* = 5) was carried out using RNAeasy kit (Quiagen, Germantown, MD, USA). Isolated RNA was quantified using Nanodrop (ThermoFischer, Waltham, MA, USA) followed by DNA digestion (Quiagen Kit). The purified RNA was then reverse transcribed to cDNA using First Strand cDNA synthesis kit, Quiagen, USA. Synthesized cDNA was diluted for downstream analysis. Primers used for the study were designed using Primer-3 tool and standardized as per the routine protocol. For real-time PCR reaction, SYBER green chemistry was used as per manufacturer’s protocol (Applied Biosystems, Waltham, MA, USA) using Step One Plus instrument (Applied Biosystems, USA). Mathematical calculation for fold change in gene expression was performed as described before [25]. RPL32 was used as reference gene [25].

### 2.10. Immunoblotting

Protein isolation was carried out using tissue protein extraction reagent (T-PER, Pierce, USA). Following centrifugation, resultant supernatant was collected and quantified using BCA kit, Pierce, USA. An equal amount of protein was resolved using 10 to 12% SDS-polyacrylamide gel. The resolved gel was then transferred to a polyvinyldine difluoride membrane (Thermo Scientific, Waltham, MA, USA). Blocking of the membrane was performed using 3–5% blotto, nonfat dry milk (Santa Cruz, Dallas, TX, USA) in TBS containing 0.1% Tween-20, at room temperature for 1 h. The membrane was then incubated with primary antibody at 4 °C overnight. Following three subsequent washings, the membrane was incubated with secondary anti-Rabbit (Cell signaling, Danvers, MA, USA. Cat No: 7074) or anti-mouse (Cell signaling, Danvers, MA, USA. Cat No: 7076) antibody for 1 h at room temperature. Blots were visualized using super signal chemiluminescent substrate (Thermo Scientific, Waltham, MA, USA. Cat No: 34080). SIRT-3 antibody (Cell signaling, Danvers, MA, USA. Cat No: 5490), SIRT-1 antibody (Abcam, Cambridge, MA, USA. Cat No: ab157401), TFAM antibody (Abcam, Cambridge, MA, USA. Cat No: ab131607), GAPDH antibody (Cell signaling, Danvers, MA, USA. Cat No: 2118), and anti-acetylated lysine antibody (Cell signaling, Danvers, MA, USA. Cat No: 9441) were used for the study.

### 2.11. Immunoprecipitation

Immunoprecipitation (IP) was carried out using Dynabeads protein G IP kit (Life Technologies, Cat No: 10007D) as per the manufacturer’s instructions. An equal amount of eluted protein was resolved on the gel for assessing the acetylation status TFAM using anti-acetyl lysine antibody.

### 2.12. Electrophoretic Mobility Shift Assay

The DNA-binding activity of TFAM was assessed by electrophoretic mobility shift assay (EMSA) using the light-shift chemiluminescent EMSA kit (Pierce, Waltham, MA, USA). Complementary oligonucleotide probes containing the D-loop region of mtDNA were designed as binding motifs (TFAM-, 5-TTTCCTCCTAACTAAACCCTCTTTAC-3) and were end-labeled with biotin. The membrane was incubated with chemiluminescent substrate and developed using a CCD camera ChemiXRS, a chemiluminescent instrument (Bio-Rad, Hercules, CA, USA). Unlabeled oligos were added to the reaction at five- to ten-fold excess to evaluate the specificity.

### 2.13. Statistical Analysis

All values are expressed as the mean ± standard error (SE). One-way analysis of variance (ANOVA) test followed by Bonferoni’s correction was carried out to test any differences between the mean values of all groups. Significance in group differences was assumed if *p* < 0.05.

## 3. Results

### 3.1. Serum Biochemistry

Rats injected with STZ showed increased blood glucose levels after eight weeks compared to CON group (Table 1). In addition, there was an increased blood HbA1c levels in DIA group rats (Table 1). Administration of resveratrol decreased the levels of blood glucose and glycated hemoglobin compared to DIA group rats. Moreover, there were reduced levels of serum insulin in DIA group rats compared to CON group rats, which increased after resveratrol administration (Table 1). Increased levels of serum SGOT in DIA group rats was also reduced after resveratrol administration (Table 1). Increased levels of serum triglyceride, uric acid, and creatinine along with reduced levels of cholesterol and HDL was observed in DIA group. Administration of resveratrol normalized all these serum metabolites alteration (Table 1). HOMA analysis showed reduced beta cell efficiency which was improved by resveratrol administration (Table 1).

### 3.2. Cardiac Atrophy and Fibrosis

**Figure 1:**
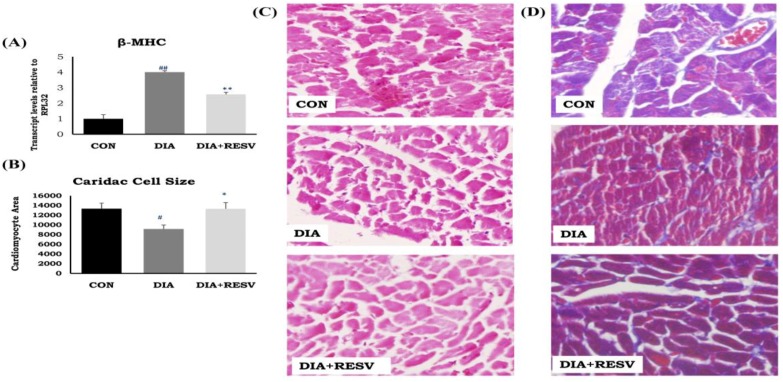
**Cardiac phenotypes and histopathological changes in diabetic rat heart and effect of resveratrol administration**. (**A**) mRNA expression of β-MHC; (**B**) Cardiac cell size; (**C**) Hematoxylin and eosin staining of rat heart tissue; (**D**) Masson’s trichrome staining of rat heart tissue. Data shown as Mean ± SE, (*n* = 6) # *p* < 0.05, ## *p* < 0.01 vs. Con; * *p* < 0.05, ** *p* < 0.01 vs. DIA group. Three sections were observed for histopathology examination and twenty cells per image were analyzed for cell size measurement.

We have previously reported that the reduced ratio of heart weight to tail length in the DIA group was increased with resveratrol administration [14]. Histopathological examination revealed reduced cell size along with significant induction of cardiac fibrosis. Increased mRNA expression of β-MHC in DIA group was observed. Administration of resveratrol significantly ameliorated all of these cardiac alterations. However, there was a significant difference in mRNA expression of β-MHC between CON and resveratrol treated group (Figure 1).

### 3.3. Mitochondrial Citrate Synthase, β-Hydroxy Acyl Co-A Dehydrogenase Activity, and Mitochondrial Number

**Figure 2:**
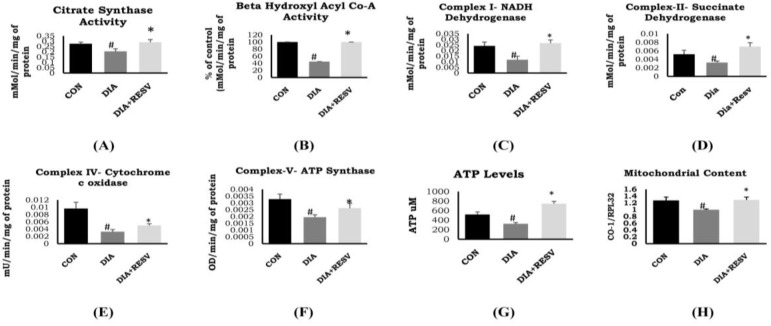
**Mitochondrial metabolic enzymes activity in diabetic rat heart and effect of resveratrol administration**. (**A**) Citrate synthase; (**B**) β-hydroxy acyl CoA dehydrogenase; (**C**) Complex-I: NADH dehydrogenase; (**D**) Complex-II: Succinate dehydrogenase; (**E**) Complex-IV: Cytochrome c oxidase; (**F**) Complex-V: ATP synthase; (**G**) ATP levels; (**H**) Mitochondrial content. Data shown as mean ± SE, (*n* = 6) # *p* < 0.05 vs. Con; * *p* < 0.05 vs. DIA group.

There was reduced activity of citrate synthase in DIA group rat heart compared to CON group rat heart (Figure 2A). In addition, there was reduced activity of β-hydroxy acyl CoA dehydrogenase in DIA group rats. Administration of resveratrol rescued the activity of citrate synthase and β-hydroxy acyl CoA dehydrogenase (Figure 2A,B). Analysis of cardiac mitochondrial content showed reduced number of mitochondria in DIA group compared to CON group. Administration of resveratrol prevented the decline in mitochondrial content compared to DIA group (Figure 2H). 

### 3.4. Activity of ETC Complex Assembly and ATP Generation

Diabetic heart showed reduced activity of complexes I, II, and IV along with reduced levels of ATP generation compared to the CON group (Figure 2). Administration of resveratrol prevented decline in the activity of complexes I, II, IV, V, and ATP content compared to the DIA group. In addition, ATP level was significantly higher in the resveratrol-treated group as compared to the CON group (Figure 2).

### 3.5. Reactive Oxygen Species Levels and Antioxidant Defense

Increased levels of ROS and TBARS in DIA group compared to CON group were observed (Table 2). In addition, there was reduced activity of SOD, catalase, and DPPH along with reduced levels of glutathione (GSH) in the DIA group compared to the CON group (Table 2). Administration of resveratrol prevented the increase in ROS levels as well as the decline in SOD, catalase, DPPH activity, and levels of GSH (Table 2).

### 3.6. mRNA Expression of Mitochondrial Encoded Genes

**Figure 3:**
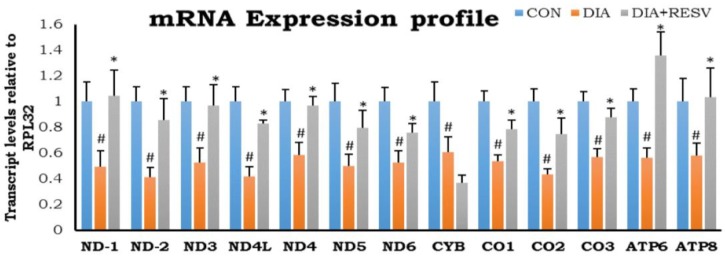
**mRNA expression profile of mitochondrial encoded genes in diabetic heart and effect of resveratrol administration**. Data shown as mean ± SE, (*n* = 5) # *p* < 0.05 vs. Con; * *p* < 0.05 vs. DIA group.

Reduced expression of all thirteen mitochondrial encoded genes in DIA group hearts compared to CON group hearts was observed. Administration of resveratrol restored the expression of all these genes except MT-CYB. In addition, mitochondrial CYB and CO-1 gene expression was significantly low in resveratrol treated group as compared to CON (Figure 3).

### 3.7. Expression and Activity of TFAM with Increased Acetylation Status

**Figure 4:**
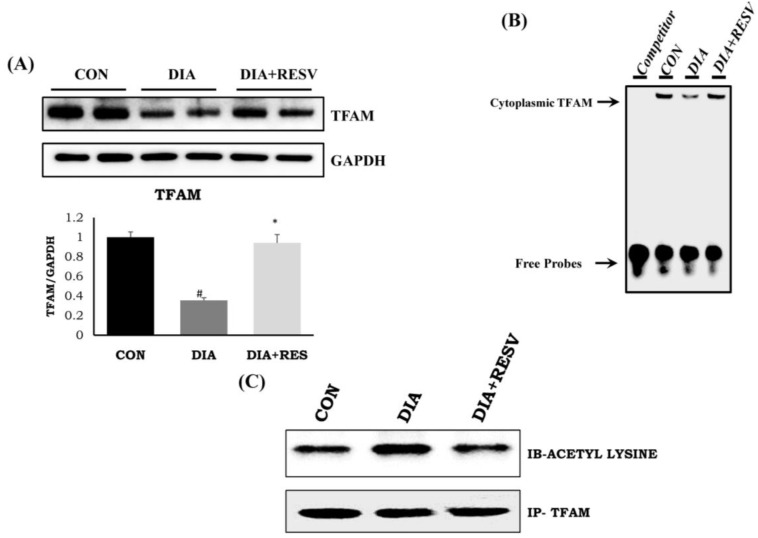
**Expression and activity of TFAM with acetylation status and effect of resveratrol administration**. (**A**) Protein expression of TFAM; (**B**) Activity of TFAM by electrophoretic mobility shift assay (EMSA) assay; (**C**) Acetylation status of TFAM. Data shown as mean ± SE, (*n* = 4) # *p* < 0.05 vs. Con; * *p* < 0.05 vs. DIA group.

Reduced expression and activity of TFAM in diabetic heart compared to CON group heart was observed (Figure 4A,B). Resveratrol administration improved the expression and activity of TFAM compared to DIA group heart. Transcript level of TFAM mRNA was increased with SIRT-3 overexpression in H9C2 cells (Supplementary Figure S1C). In addition, there was increased acetylation status of TFAM in DIA group heart compared to CON group heart. However, this increased acetylated TFAM levels was reduced with resveratrol administration (Figure 4C).

### 3.8. Expression and Activity of SIRT-1 and SIRT-3

**Figure 5:**
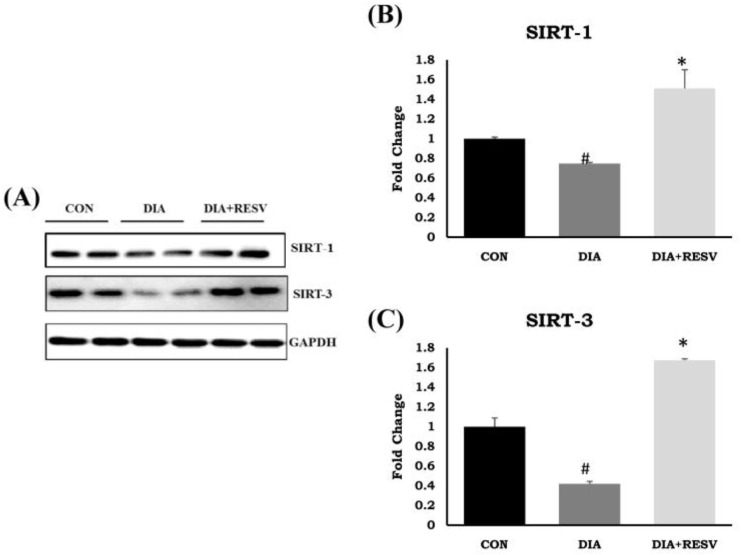
**Expression of SIRT-1 and SIRT-3 in diabetic rat heart and effect of resveratrol administration**. (**A**) Immunoblotting of SIRT-1 and SIRT-3; (**B**,**C**) Fold change in SIRT-1 and SIRT-3 protein expression. Data shown as mean ± SE, (*n* = 5) # *p* < 0.05 vs. Con; * *p* < 0.05 vs. DIA group.

The reduced expression and activity of SIRT-1 and SIRT-3 in type-I diabetic heart compared to the CON group heart has already been reported by us. Administration of resveratrol prevented the decline in expression and activity of both SIRT-1 and SIRT-3 in diabetic heart. However, SIRT-1 and SIRT-3 protein expression was significantly higher in the resveratrol treated group compared to the CON group (Figure 5) [14].

### 3.9. TFAM Acetylation Is Regulated by SIRT-3

**Figure 6:**
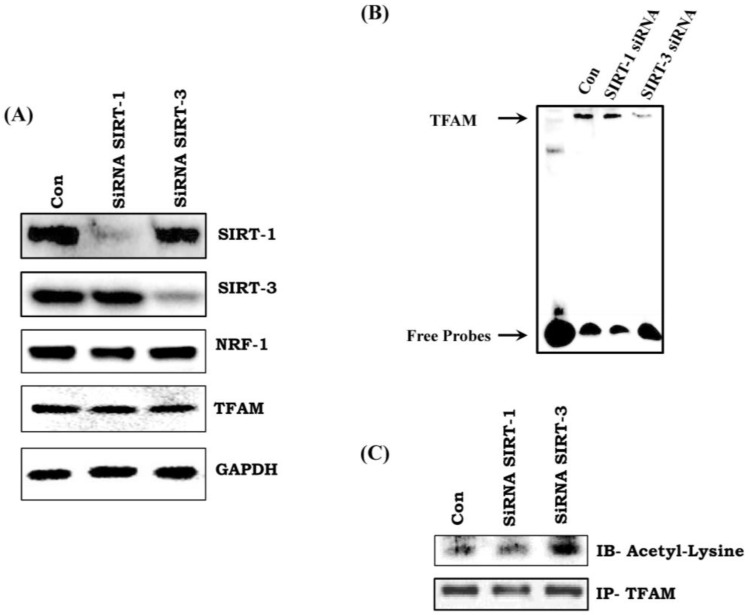
**SIRT-3 specifically regulates the acetylation status of TFAM.** (**A**) Effect of SIRT-1 and SIRT-3 silencing on TFAM and NRF-1 expression; (**B**) TFAM activity as measured by EMSA assay in SIRT-1 and SIRT-3 silenced cells; (**C**) Acetylation status of TFAM in SIRT-1 and SIRT-3 silenced cells. Data shown as representative of three different experiments.

There was no change in the expression of TFAM and NRF-1 in SIRT-1 and SIRT-3 silenced H9C2 cells (Figure 6A). In addition, we did not observe any change in the mRNA transcript level of NRF-1 and TFAM in SIRT-3 (shRNA) silenced H9C2 cells (Supplementary Figure S1D,E). However, there was reduced activity of TFAM in SIRT-3 silenced cell, but not in SIRT-1 silenced cells (Figure 6B). Immunoprecipitation followed by immunoblotting with anti-acetyl lysine of TFAM showed increased acetylation status of TFAM in SIRT-3 silenced cells (Figure 6C). Thus, our data indicates that lack of SIRT-3 reduces TFAM activity via hyperacetylation.

## 4. Discussion

Diabetes is a complex metabolic disease that leads to cardiac complications as the disease progresses. Hyperglycemia induces multiple molecular and phenotypic perturbations, leading to the development of cardiac dysfunction [26]. Although the incidence of T1DM is only 5 to 10% in the diabetic populace, its severity is comparatively higher than type II diabetes mellitus. Hyperglycemia remains the common culprit in both types of diabetes and contributes most to the pathology of cardiac complications. In the present study, we have selected the T1DM rat model to study the effect of hyperglycemia and lack of insulin on cardiac mitochondrial energetics and transcription.

Our study showed increased blood glucose and reduced serum insulin levels in STZ injected rats. The hyperglycemic condition sustained throughout the study duration, as indicated by increased HbA1c levels. In addition, there was increased level of serum triglyceride, serum creatinine, uric acid, and SGOT in diabetic rats along with reduced levels of serum HDL and total cholesterol. All these altered metabolite profiles were normalized with resveratrol administration. Thus all the above serum metabolite changes indicate the development of T1DM, which is a well-known phenomenon in this model [27].

Cardiac complications in this T1DM model revealed that there was reduced heart weight to tail length ratio, called cardiac atrophy. This was supported by histopathological examination and a reduction in cardiac cell size. We observed the incidence of cardiac atrophy in T1DM, contrast with T2DM, where cardiac hypertrophy is the predominant phenotype [28]. Previous reports also showed similar outcomes and indicate the effect of reduced insulin and starved condition [29,30,31]. Administration of resveratrol prevented reduction in the cell size as well as heart weight to tail length ratio. Moreover, the reduced cardiac cell size may be one of the reasons of increased apoptosis in cardiomyocytes as described before [30]. These dead cells are then replaced by fibrosis as a natural repair mechanism, which we have observed in diabetic rat heart in our present study.

We have observed the reduced mitochondrial activity of citrate synthase and β-hydroxyacyl coA dehydrogenase in diabetic rat heart. In addition, there was reduced activity of ETC complex assembly and ATP levels. All these outcomes may be associated with reduced cardiac size because of deficient mitochondrial energetics. Along with these changes, we have also observed reduced cardiac mitochondrial content in diabetic rats. Administration of resveratrol ameliorated these reduced enzyme activities, along with increasing the levels of ATP and mitochondrial content. Data showed that resveratrol improved cardiac phenotype with increased mitochondrial efficiency and content. Increased SIRT3 expression and activity in the diabetic heart after resveratrol administration might be responsible for improved mitochondrial ETC activity. Although, we have not explored the exact mechanism of SIRT-3 activation by resveratrol in this study, previous work showed the involvement of estrogen-related receptor alpha (ERRα) for SIRT-3 expression [32,33]. Apart from regulating mitochondrial energy metabolism, SIRT-3 plays a critical role in attenuating cardiac fibrosis. Chen et al. recently showed that activation of SIRT-3 by resveratrol improved cardiac function and attenuated cardiac fibrosis by inhibiting TGF β/Smad pathway [15]. We also observed increased fibrosis in diabetic rat heart in our present study. Administration of resveratrol reduced cardiac fibrosis in diabetic rat heart. SIRT-3 also plays a critical role in regulating autophagy during diabetes cardiomyopathy. Previously, it has been shown that resveratrol promotes autophagy by upregulating SIRT-3 expression and phosphorylating AMP-activated protein kinase (AMPK) [34]. However, in the present study, we are more focused on understanding the regulation of cellular metabolism by SIRT-3 in mitochondria.

We evaluated whether the improvement of mitochondrial efficiency through resveratrol is due to enhancement of mitochondrial biogenesis. TFAM, a mitochondrial transcription factor, is involved in the transcriptional control of mtDNA, whereas NRF-1 is involved in the control of nuclear DNA transcription for the mitochondrial proteins. We have observed reduced expression and activity of TFAM with no change in NRF-1 (data not shown) in diabetic heart. This suggests that the nuclear trigger for mitochondrial biogenesis is not affected in this diabetic heart and the problem persists mostly at the mitochondrial levels. As TFAM is the only mitochondrial transcription factor involved in transcription of mtDNA that encodes proteins for ETC complex, reduced activity of the ETC complex and its activity is expected in diabetic heart. We have also tried to elucidate if the altered TFAM activity is due to reduced expression and activity of SIRT-1 and SIRT-3 in diabetic heart as observed in the present study and previously [14]. SIRT-1 is involved in deacetylating PGC-1α, which regulates the action of NRF-1 as reported earlier [35,36]; whereas, SIRT-3, a major mitochondrial deacetylase, controls mitochondrial health [37]. To find out the underlying mechanism of the regulation of TFAM activity, we have silenced SIRT-1 and SIRT-3 in H9C2 cells. We found no change in the expression of TFAM and NRF-1. However, there was reduced activity of TFAM in SIRT-3 silenced cells but not in SIRT-1 silenced cells. Next, we demonstrated whether SIRT-3 controls the acetylation status of TFAM and its activity. We found that silencing SIRT-3 increases the acetylation of TFAM and reduces its DNA binding activity, which was not observed in SIRT-1 silenced cells. This reinforces that SIRT-3 is crucial in regulating the acetylation status of TFAM and also its activity. As observed in in vitro cell line, we have also observed the acetylation status of TFAM in diabetic rat heart in vivo. Reduced activity of SIRT-3 in diabetic rat heart was associated with increased acetylation of TFAM, which leads to reduced activity. Administration of resveratrol prevented the decline in SIRT-3 activity and TFAM acetylation as well as its activity in diabetic rat heart.

## 5. Conclusions

In conclusion, we have observed that reduced SIRT-3 activity in diabetic heart is associated with increased acetylation and reduced activity of TFAM, which corresponds to reduced activity of ETC complex assembly and ATP levels and decreased cardiac size. Administration of resveratrol prevented all these cardiac changes and improves cardiac health. Our data indicates the potential role of SIRT-3 in Type-I diabetic heart providing an opportunity for future therapeutic intervention.

## Figures and Tables

**Table 1:** Serum biochemistry of diabetic and resveratrol administered rats.

Parameters	CON	DIA	DIA + RESV
Blood glucose, mg/dL	88.58 ± 2.90	429.00 ± 22.53 ^##^	217.50 ± 37.83 **
Glycated Hemoglobin, %	5.84 ± 0.16	9.71 ± 0.13 ^##^	7.46 ± 0.15 *
Insulin, pmol	131.01 ± 15.74	44.87 ± 6.70 ^##^	97.32 ± 8.26 *
Triglyceride, mg/dL	116.87 ± 8.255	157.00 ± 15.38 ^#^	122.60 ± 6.47 *
Cholesterol, mg/dL	72.77 ± 3.55	53.00 ± 2.58 ^#^	74.66 ± 5.40 **
Uric acid, mg/dL	0.85 ± 0.034	1.44 ± 0.15 ^#^	0.92 ± 0.05 *
Creatinine, mg/dL	0.36 ± 0.01	0.52 ± 0.03 ^#^	0.26 ± 0.01 *
HDL, mg/dL	62.71 ± 1.02	48.20 ± 2.09 ^#^	64.80 ± 3.69 *
HOMA IR	4.00 ± 0.46	26.65 ± 8.25 ^#^	5.52 ± 1.18 **
HOMA %B	278.70 ± 19.15	13.61 ± 2.56 ^#^	58.20 ± 17.59 *
HOMA %S	28.12 ± 3.07	15.46 ± 5.95 ^#^	20.60 ± 3.76 *

Data are the mean ± SE of individual data sets. ^#^ *p* < 0.05; ^##^ *p* < 0.01 vs. Conl and * *p* < 0.05; ** *p* < 0.01; vs. DIA (*n* = 8). Part of the data is reprinted from Biochemical and Biophysical Research Communications, 468, Bagul PK, Dinda AK, Banerjee SK, “Effect of resveratrol on sirtuins expression and cardiovascular complications in diabetes” 221–227, 2015, with permission from Elsevier.

**Table 2:** Cardiac antioxidant and oxidative stress markers in diabetic rats and effect of resveratrol administration.

Parameters	CON	DIA	DIA + RESV
TBARS (uM/ gm weight of tissue)	559.57 ± 26.44	707.53 ± 26.32 ^#^	576.29 ± 4.09 **
ROS (%)	100	160.18 ± 1.87 ^##^	95.24 ± 1.07 **
DPPH (% scavenging)	26.68 ± 2.30	18.61 ± 1.43 ^#^	27.86 ± 5.01 *
GSH (ng/mg of protein)	398.23 ± 6.00	378.67 ± 5.34 ^#^	417.50 ± 12.58 *
Catalase (mU/ug of protein)	1.85 ± 0.11	1.04 ± 0.28 ^#^	3.26 ± 0.52 **
SOD (% activity)	100	68.84 ± 2.53 ^##^	81.51 ± 3.62 *

Data are the mean ± SE of individual data sets. ^#^ *p* < 0.05; ^##^ *p* < 0.01 vs. CON and * *p* < 0.05; ** *p* < 0.01; vs. DIA (*n* = 8).

## Supplementary Materials

The following are available online at http://www.mdpi.com/2073-4409/7/12/235/s1, Figure S1: Effect of SIRT-3 overexpression and stable knockdown in H9c2 cells.
