# Supplementary material for: SIRT-3 Modulation by Resveratrol Improves Mitochondrial Oxidative Phosphorylation in Diabetic Heart through Deacetylation of TFAM
Source: Cells. 2018 Nov 28;7(12):235. doi: 10.3390/cells7120235 (PMC6315986; doi:10.3390/cells7120235)
Supplement: Supplementary file 1 [file cells-07-00235-s001.pdf]

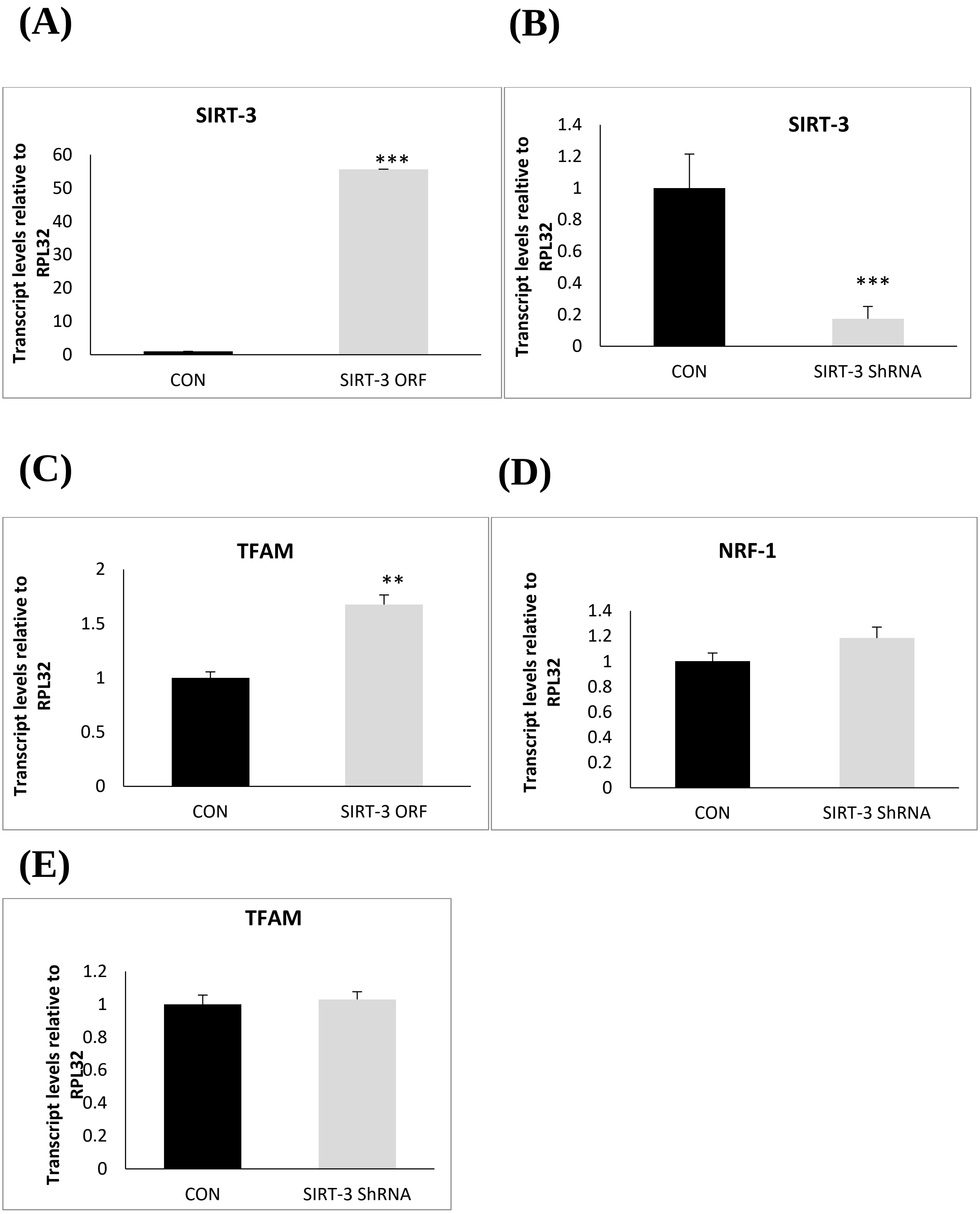

Supplementary. Effect of SIRT-3 overexpression and stable knockdown in H9c2 cells. A) mRNA expression of SIRT-3 after SIRT-3 overexpression in H9c2 cells. B) mRNA expression of SIRT-3 after SIRT-3 silencing using shRNA in H9c2 cells. C) mRNA expression of TFAM after SIRT-3 over expression in H9c2 cells. D) mRNA expression of NRF-1 after SIRT-3 silencing using shRNA in H9c2 cells. E) mRNA expression of TFAM after SIRT-3 silencing using shRNA in H9c2 cells.
